# The cytotoxic action of BCI is not dependent on its stated DUSP1 or DUSP6 targets in neuroblastoma cells

**DOI:** 10.1002/2211-5463.13418

**Published:** 2022-05-06

**Authors:** Elliott M. Thompson, Vruti Patel, Vinothini Rajeeve, Pedro R Cutillas, Andrew W. Stoker

**Affiliations:** ^1^ Developmental Biology & Cancer Research and Teaching Department UCL Great Ormond Street Institute of Child Health London UK; ^2^ Mass Spectrometry Laboratory Barts Cancer Institute Queen Mary University of London UK; ^3^ Present address: Current Address: Discovery Research MRL UK MSD The London Bioscience Innovation Centre (LBIC) London UK

**Keywords:** chemical inhibition, CRISPR‐Cas9, dual‐specificity phosphatase, MAPK signalling, neuroblastoma, phosphoproteomics

## Abstract

Neuroblastoma (NB) is a heterogeneous cancer of the sympathetic nervous system, which accounts for 7–10% of paediatric malignancies worldwide. Due to the lack of targetable molecular aberrations in NB, most treatment options remain relatively nonspecific. Here, we investigated the therapeutic potential of BCI, an inhibitor of DUSP1 and DUSP6, in cultured NB cells. BCI was cytotoxic in a range of NB cell lines and induced a short‐lived activation of the AKT and stress‐inducible MAP kinases, although ERK phosphorylation was unaffected. Furthermore, a phosphoproteomic screen identified significant upregulation of JNK signalling components and suppression in mTOR and R6K signalling. To assess the specificity of BCI, CRISPR‐Cas9 was employed to introduce insertions and deletions in the DUSP1 and DUSP6 genes. Surprisingly, BCI remained fully cytotoxic in NB cells with complete loss of DUSP6 and partial depletion of DUSP1, suggesting that BCI exerts cytotoxicity in NB cells through a complex mechanism that is unrelated to these phosphatases. Overall, these data highlight the risk of using an inhibitor such as BCI as supposedly specific DUSP1/6, without understanding its full range of targets in cancer cells.

## Graphical abstract

This study examined whether the cytotoxic effects of BCI on neuroblastoma tumour cells were exerted by its known inhibitory action on dual specificity phosphatases DUSP1 and DUSP6. We demonstrated that this was not the case and that BCI elicits cytotoxicity through off‐target effects, potentially through the activation of MTOR and JNK pathways.

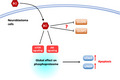

**Abbreviations**
BCI: (E/Z)‐BCI hydrochloride
DUSP: dual‐specificity phosphatase
indels: insertion deletions
KSEA: kinase substrate enrichment analysis
MAPK: mitogen‐activated protein kinase
NB: neuroblastoma
PTP: protein tyrosine phosphatase
ROS: reactive oxygen species

Neuroblastoma (NB) is the most prevalent extracranial paediatric cancer and accounts for 7–10% of childhood cancers worldwide [1]. Tumours arise during the development of the sympathetic nervous system and manifest predominantly in the sympathetic ganglia and adrenal glands [2]. Patients are stratified into various risk groups based on a range of clinical and pathological features. Unfortunately, half of the patients develop high‐risk tumours and the 5‐year survival of these patients remains below 50% [2, 3, 4]. Due to the heterogeneous nature of the disease and lack of targetable aberrations, treatment options remain relatively nonspecific [5]. Hence, there is an urgent need to identify the molecular pathways that drive NB cell growth, against which specific therapeutics can be developed.

The dual‐specificity phosphatase family (DUSPs) represents a large group of protein tyrosine phosphatases (PTPs) that can dephosphorylate both Tyr and Ser/Thr residues [6]. DUSPs regulate a range of biological processes as they restrain various kinase signalling cascades. This is especially true for the MAP kinase phosphatases, a subset of evolutionarily conserved DUSPs that display high specificity to the MAP kinases [7]. Various DUSPs have been identified as potential therapeutic targets for NB including DUSP1, DUSP8, DUSP10 and DUSP16 as they suppress the pro‐apoptotic JNK and p38 kinases [8]. For example, DUSP1 overexpression not only inhibited JNK signalling in N1E115 cells under hypoxic conditions but also the expression of downstream apoptotic genes and neuronal cell death [9]. Although logically the ERK‐specific DUSPs should function as tumour suppressors by restraining oncogenic ERK signalling, this is not always the case. In mouse embryonic fibroblasts oncogenically transformed with HER2, silencing of DUSP6 prevents cell growth and triggers apoptosis [10]. Similarly, in melanoma cells containing the BRAF V600E mutations, loss of DUSP6 induces apoptotic signalling [11]. This suggests that in the context of constitutive ERK activation, reducing DUSP6 expression potentially triggers ERK‐mediated apoptosis as opposed to increased cell proliferation. Considering a subpopulation of NB tumours is maintained by ERK signalling via the ALK oncogene, manipulation of DUSP6 activity might suppress NB cell growth [12].

Protein tyrosine phosphatases contain a highly conserved active site. It is therefore very challenging to generate specific phospho‐mimetic inhibitors. Nonetheless, after prolonged efforts, various phosphatase inhibitors have been generated that are now in clinical trials [13, 14]. To date, only one DUSP inhibitor has been identified that demonstrates high target selectivity. Identified in an *in vivo* chemical screen, (E/Z)‐BCI hydrochloride (BCI) is a dual‐inhibitor of DUSP1 and DUSP6 [15]. Molecular docking simulations demonstrated BCI bound to the low‐activity conformation of DUSP6 in an allosteric manner and prevented ERK‐mediated activation of DUSP6 [16]. Promisingly, BCI did not affect DUSP5 function in a transgenic zebrafish model and did not inhibit recombinant CDC25B, PTP1B or DUSP3 in vitro [15], suggesting some specificity towards DUSP1 and DUSP6. Furthermore, phosphokinase profiling of a second‐generation BCI analogue, BCI‐215, demonstrated selective activation of the mitogen‐activated protein kinase (MAPK) pathways, suggesting minimal off‐target toxicities [17]. BCI also demonstrates anti‐tumorigenic activity in a range of cancer models, it is suggested predominantly via inhibition of DUSP6 [18, 19, 20]. In SH‐SY5Y NB cells, treatment with BCI led to a dose‐dependent decrease in ERK activity and cell viability, hinting that pharmacological inhibition of DUSP1 and DUSP6 could potentially reduce NB cell growth [21]. In our present study, we assessed the cytotoxicity of BCI and BCI‐215 across a range of NB cell lines and investigated their biochemical influence over DUSP1/DUSP6‐related signalling pathways. A broad phosphoproteomic screen was also performed to identify the signalling pathways responsible for BCI‐mediated cell death. Lastly, insertion deletions (indels) were introduced into the DUSP1 and DUSP6 genes to test for any off‐target effects of BCI. Consequently, we made the surprising finding that although BCI and BCI‐215 are cytotoxic, the depletion of either DUSP1 or DUSP6, or both, did not affect the cells’ cytotoxic response to the chemicals. Collectively, these data suggest that BCI and BCI‐215 have toxic activities unrelated to their DUSP1/6 enzyme targets in several NB cell lines and thus cannot be recommended as reliable DUSP1/6 inhibitors in this context.

## Materials and methods

### Cell culture and chemicals

KELLY and SK‐N‐AS cells were a gift from Prof. Frank Speleman, University of Ghent (STR genotyped). IMR‐32 was from ATCC and LAN‐1 was a gift from Prof. John Anderson, UCL, London. Cells have been STR tested. Cells were grown at 37 °C and 5% C0_2_ in a humidified incubator. All cell lines were cultured in RPMI medium + GlutaMAX™ (ThermoFisher Scientific, Loughborough, UK) supplemented with 10% FBS, 100 U·mL^−1^ Penicillin, 0.1 mg·mL^−1^ Streptomycin and 25 mm HEPES. Cells were routinely checked for mycoplasma using the MycoAlertTM PLUS Mycoplasma Detection Kit (Lonza, Slough, UK). BCI (317496) was purchased from ThermoFisher Scientific and SP600125 (sc‐200635) and SB202190 (sc‐202334) were purchased from Santa Cruz Biotechnology (Heidelberg, Germany). BCI‐215 was a gift from Prof. Andreas Vogt (University of Pittsburgh, USA). Chemical treatments were performed 24 h after the seeding of cells.

### Plasmid transfection

Cells were seeded in 12‐well plates at 50% confluency. The following day, cells were transiently transfected with plasmids after incubation with Lipofectamine 2000 (ThermoFisher Scientific) for 5 min. Transfections included pEGFP‐N2 (Clontech, Mountain View, CA, USA) for quantifying transfection efficiency.

### Generation of DUSP6 knockout subclones

To deplete DUSP6 protein, an inducible CRISPR system was implemented where Cas9 expression was under the control of a doxycycline‐inducible promoter in a lentiviral vector (Horizon Discovery, Cambridge, UK; #VCAS11227). The lentiviral vector was transduced into IMR‐32 and KELLY cells and selected with blasticidin to generate inducible, clonal populations called iIMR‐32 and iKELLY. Following this, the DUSP6 gRNA (Table 1) was inserted into a pU6 sgRNA vector (Addgene, Watertown, MA, USA; #60955) and transfected into iIMR‐32 and iKELLY cells. Cells were selected with puromycin and incubated for 4 weeks allowing stable selection of Cas9‐inducible clones expressing the gRNA plasmid. These cells were treated with 0.5 μg·mL^−1^ Dox and incubated for 5–7 days allowing induction of Cas9 and editing of the DUSP6 gene. Individual subclones were then expanded. KELLY and IMR‐32 subclones generated with complete loss of DUSP6 protein were termed KELD6‐1, KELD6‐2 and IMRD6‐1, respectively.

**Table 1:** DUSP1 and DUSP6 gRNA sequences.

Gene	gRNA sequence
DUSP1 gRNA	TCAGGGACGCTAGTACTCAG
DUSP6 gRNA	CATCGAGTCGGCCATCAACG

### Generation of DUSP1 mixed populations

A vector that co‐expressed Cas9 and DUSP1 gRNA was purchased from VectorBuilder (Neu‐Isenberg, Germany) (Table 1). IKELLY cells and DUSP6‐null subclones were transfected with this DUSP1 vector or a control pU6 sgRNA vector (Addgene, Watertown, MA, USA; #60955). Cells were incubated for 3 days to generate indels in the DUSP1 gene. Subsequently, genomic DNA was extracted and sent for sequencing to determine the frequency of indels in the mixed populations. Our guide RNAs were also designed to target highly conserved protein regions, using snap2 software [22], in order to maximise detrimental protein disruption when indels are in‐frame. Two thousand cells of the mixed DUSP1 indel populations were seeded into 96‐well plates. After 24 h, cells were treated with varying concentrations of BCI and a cell viability assay was performed after a 6‐day incubation.

### Sequencing of DUSP1 and DUSP6 guide RNA‐treated subclones

The indels present in DUSP6 subclones and DUSP1 mixed populations were characterised by DNA sequencing. Genomic DNA was extracted from cells using QuickExtract™ DNA Extraction Solution (Cambio, Cambridge, UK). Regions containing the edited site were amplified via PCR (Primers given in Table 2) and samples subjected to Sanger sequencing (Source Bioscience, Nottingham, UK). Sequence trace analysis was performed using the ice analysis online tool (Synthego, v2.0; Synthego, Menlo Park, CA, USA).

**Table 2:** Primers designed to either side of DUSP1 and DUSP6 gRNA site.

Gene	Primers
DUSP1‐FWD	5’‐CTCAAAGGTACGCCCTCGG‐3’
DUSP1‐REV	5’‐GGAGCTGATGTCTGCCTTGT‐3’
DUSP6‐FWD	5’‐TGAGACGCTCGCTGTTTGTA‐3’
DUSP6‐REV	5’‐CTGAGCGGAGCAGAGGTATT‐3’

### Cell viability assays

The resazurin salt assay was performed to measure cell viability. 2 × 10^3^ SK‐N‐AS, KELLY and IMR‐32 cells and 1 × 10^3^ LAN‐1 cells were seeded in a 96‐well plate. Chemical treatments were performed after 24 h and cells were then incubated for 6 days. Following this, resazurin (Merck Life Science UK Ltd, Gillingham, UK) dissolved in PBS was added to cells at a final concentration of 0.0015% w/v. Cells were incubated for 4 h and fluorescence intensity was read on a FLUOstar OPTIMA microplate reader (BMG Labtec Ltd., Aylesbury, UK). Data from synergy assays were analysed using Synergyfinder 2.0 [23].

### Western blotting

Cells were lysed on ice for 30 min in: 50 mm Tris Base pH 7.6; 150 mm NaCL; 1% Triton X‐100; 0.02% Sodium Azide; 1 mm protease inhibitor cocktail (Merck Life Science UK Ltd); 1 mm sodium orthovanadate; 25 mm sodium fluoride. Protein concentration was determined using the Bradford Assay (Bio‐Rad, Watford, UK). Proteins were resolved by SDS‐PAGE using 10% polyacrylamide gels and transferred to PVDF membranes. Membranes were blocked in TBS (15.4 mm Trizma‐HCL; 4.62 mm Tris‐base, 150 mm NaCl; pH 7.6) containing 10% w/v of milk powder (Merck Life Science UK Ltd). Membranes were incubated with primary antibody overnight and then with secondary antibodies conjugated to HRP (Agilent Technologies, Stockport, UK) for 1 h. Primary antibodies were from Cell Signalling (Leiden, The Netherlands): pERK (#9106; 1 : 1000); tERK (#9102; 1 : 2000); pAKT (#4060; 1 : 1000); tAKT (#9272; 1 : 2000); pp38 (#9211; 1 : 1000); tp38 (#9212; 1 : 2000); pJNK (#4668; 1 : 1000); tJNK (#9252; 1 : 2000); GAPDH (#2118; 1 : 10 000); cleaved caspase‐3 antibody (#9661, 1:250). Other primary antibodies were from Abcam (Cambridge, UK): DUSP6 (ab76310; 1 : 2000); Cas9 (ab189380; 1 : 5000). HRP was detected using ECL Prime (Cytiva, Amersham, UK) and immunoblots were quantified using image studio lite (v5.2; LI‐COR, Lincoln, NE, USA). To strip and re‐probe membranes, membranes were incubated in 0.2 m NaOH for 20 min at 37 °C and then 20 min at room temperature before re‐blocking and re‐use.

### Immunofluorescence

Prior to immunostaining, KELLY cells were plated for 24 h onto glass coverslips precoated with poly‐l‐lysine (MP Biomedicals, Eschwege, Germany; MW 30 000–70 000; 0.5 mg·mL^−1^ solution in H_2_0). After treatment with BCI, coverslips were washed in PBS three times, fixed in 4% paraformaldehyde for 30 min and blocked in PBS containing 1% BSA w/v and 0.05% Triton for 1 h. Coverslips were incubated in cleaved caspase‐3 antibody or rabbit IgG control (#10500C; ThermoFisher Scientific) for 1 h and then incubated in goat anti‐rabbit Alexa Fluor488‐conjugated secondary antibody (ThermoFisher Scientific; 1 : 300) containing DAPI (Severn Biotech Ltd., Kidderminster, UK) for 1 h. Primary and secondary antibodies were diluted in PBS containing 3% BSA w/v and 0.05% Triton. Coverslips were mounted using ProLong Gold (ThermoFisher Scientific) and imaged on an IX71 microscope (Olympus, Southend‐on‐Sea, UK). Five images were taken per sample and experiments were replicated three times.

### Phosphoproteomic analysis

Phosphoproteomics experiments were performed using mass spectrometry as reported [24, 25]. In brief, KELLY cells were plated subconfluently and one day later were treated with 2 µm BCI for 2 h before being lysed in 8 m urea buffer supplemented with phosphatase inhibitors [10 mm Na_3_VO_4_, 100 mm β‐glycerol phosphate and 25 mm Na_2_H_2_P_2_O_7_ (Merck Life Science UK Ltd)]. Proteins were digested into peptides using trypsin as previously described [26, 27]. Phosphopeptides were enriched from total peptides by TiO_2_ chromatography essentially as reported previously [28]. Dried phosphopeptides were dissolved in 0.1% TFA and analysed by nanoflow ultimate 3000 RSL nano instrument coupled online to a Q‐Exactive plus mass spectrometer (ThermoFisher Scientific). Gradient elution was from 3% to 28% solvent B in 90 min at a flow rate of 250 nL·min^−1^ with solvent A being used to balance the mobile phase (buffer A was 0.1% formic acid in water and B was 0.1% formic acid in acetonitrile). The spray voltage was 1.95 kV and the capillary temperature was set to 255 °C. The Q‐Exactive plus was operated in data‐dependent mode with one survey MS scan followed by 15 MS/MS scans. The full scans were acquired in the mass analyser at 375–1500 *m*/*z* with the resolution of 70 000, and the MS/MS scans were obtained with a resolution of 17 500.

MS raw files were converted into Mascot Generic Format using mascot distiller (version 2.6.1; Matrix Science, London, UK) and searched against the SwissProt database (release Sep 2018) restricted to human entries using the mascot search daemon (version 2.6.0; Matrix Science). Allowed mass windows were 10 ppm and 25 mmu for parent and fragment mass to charge values, respectively. Variable modifications included in searches were oxidation of methionine, pyro‐glu (N‐term) and phosphorylation of serine, threonine and tyrosine. All raw data are presented in Dataset S1.

Biological pathway enrichment was performed on phosphopeptides that satisfied the thresholds Log2 Fold Change > 2 and *P* value < 0.1 using the funrich software (v3.1.3) [29]. This included 209 and 439 phosphopeptides for the IMR‐32 and KELLY datasets, respectively. Kinase substrate enrichment analysis (KSEA) was performed on each dataset as described before by calculating a *z*‐score between the distribution of changes in phosphorylation of substrates for given kinases relative to the distribution of all fold changes [24]. Phosphoproteins selected for network visualisation were taken from published literature and KSEA networks and were visualised in cytoscape [30] using the stringapp [31] and omics visualiser plug‐ins [32]. The complete KSEA data output is provided in Dataset S2.

### Statistical analysis

To determine statistical significance between two groups, an independent *t*‐test was used and where multiple groups were compared, a one‐way ANOVA was used with a Dunnett *post* *hoc* analysis. Statistical analysis was performed on spss (v27; IBM, Portsmouth, UK).

## Results

### BCI and BCI‐215 cause cytotoxicity in neuroblastoma cell lines

**Fig. 1:**
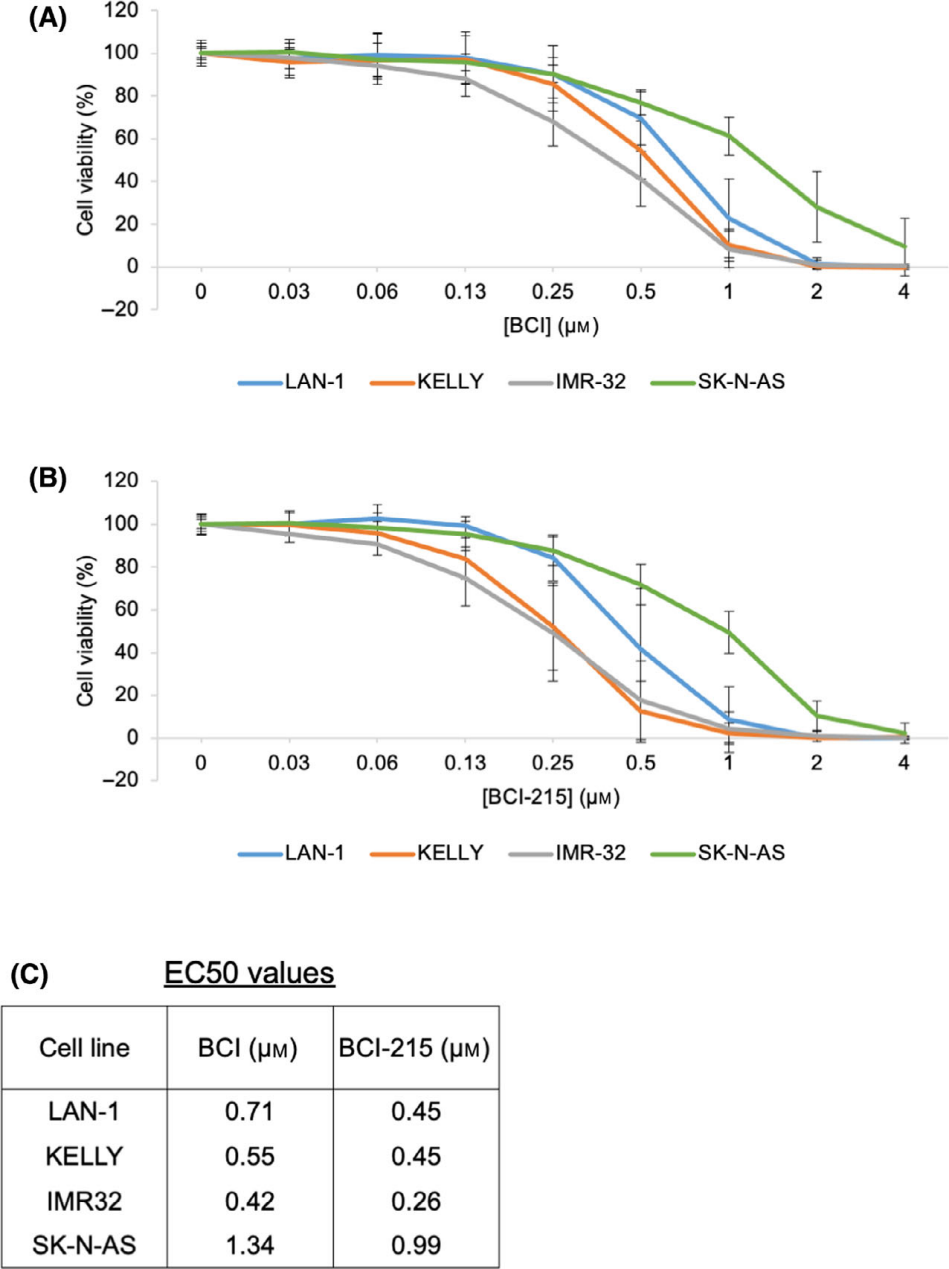
BCI and BCI‐215 are cytotoxic in a range of NB cell lines. NB cell line cells were treated with increasing doses of (A) BCI or (B) BCI‐215 followed by a cell viability assay. Data were normalised to cells treated with DMSO and then expressed as a mean ± SD (*n* ≥ 3) to generate the plotted curves. (C) Mean EC50 values were determined from the curves and are shown in the table.

Our initial aim was to determine whether BCI, an inhibitor of DUSP1 and DUSP6, was cytotoxic in NB cells. A previous study demonstrated BCI decreased the cell viability of SH‐SY5Y NB cells in a dose‐dependent manner [21]. To perform a more detailed analysis, including MYCN‐amplified lines, dose‐response assays were performed in NB cell lines SK‐N‐AS, KELLY, IMR‐32 and LAN‐1 (Fig. 1A). Recent transcriptomic profiling of NB cell lines demonstrated SK‐N‐AS cells have significantly higher expression of DUSP6 mRNA than KELLY and IMR‐32 cells and DUSP1 levels were highest in KELLY cells [33]. After 6 days of BCI treatment, BCI suppressed cell numbers in all NB cell lines, with EC50 values ranging from 0.42 to 1.34 µm (Fig. 1C). As the SK‐N‐AS cells are multi‐drug resistant, they unsurprisingly demonstrated the highest EC50 value of 1.34 µm. In parallel, dose‐response assays were performed with the BCI analogue, BCI‐215. BCI‐215 generated more growth suppression than BCI in all cell lines, with EC50 values ranging from 0.26 to 0.99 µm (Fig. 1B,C).

**Fig. 2:**
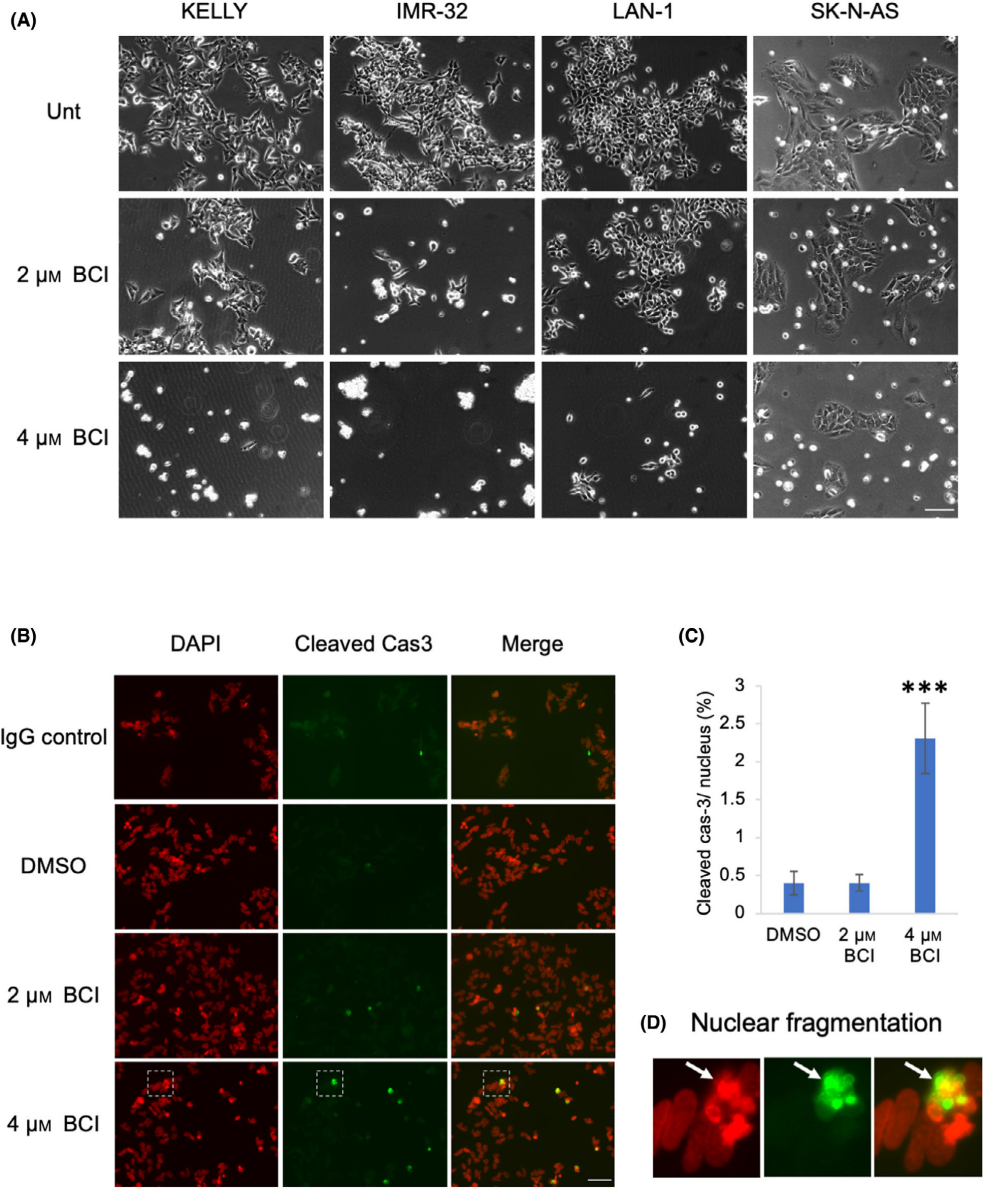
BCI triggers apoptosis in KELLY cells (A) NB cells treated with 2 or 4 µm BCI for 3 days demonstrate a dose‐dependent increase in cellular cytotoxicity. Scale bar = 50 µm. (B) KELLY cells were treated with 2 or 4 µm BCI for 24 h and then cleaved Cas3 expression was identified via immunofluorescence. Selected images are representative examples. (C) Quantification of cleaved Cas3‐positive cells reveals that 4 µm BCI increases apoptosis after 24 h (*n* = 3). One‐way ANOVA compared with DMSO; ***P ≤ 0.0005. (D) Enlargement of white dashed boxes in B demonstrates nuclear fragmentation induced by 4 µm BCI. Scale bar = 50 µm.

Observations made after 3 days of treatment showed that 4 µm BCI already elicited extensive cytotoxicity in KELLY and IMR‐32 cells, and only a minority of LAN‐1 and SK‐N‐AS cells remained. This demonstrates that BCI induces cytotoxicity in NB cells as opposed to a cytostasis (Fig. 2A). Treatment with 4 µm BCI resulted in a significant increase in the proportion of cells expressing cleaved caspase‐3, demonstrating activation of apoptosis (Fig. 2B,C). This is accompanied by nuclear fragmentation, which is visible after 24 h (Fig. 2D, white arrows).

**Fig. 3:**
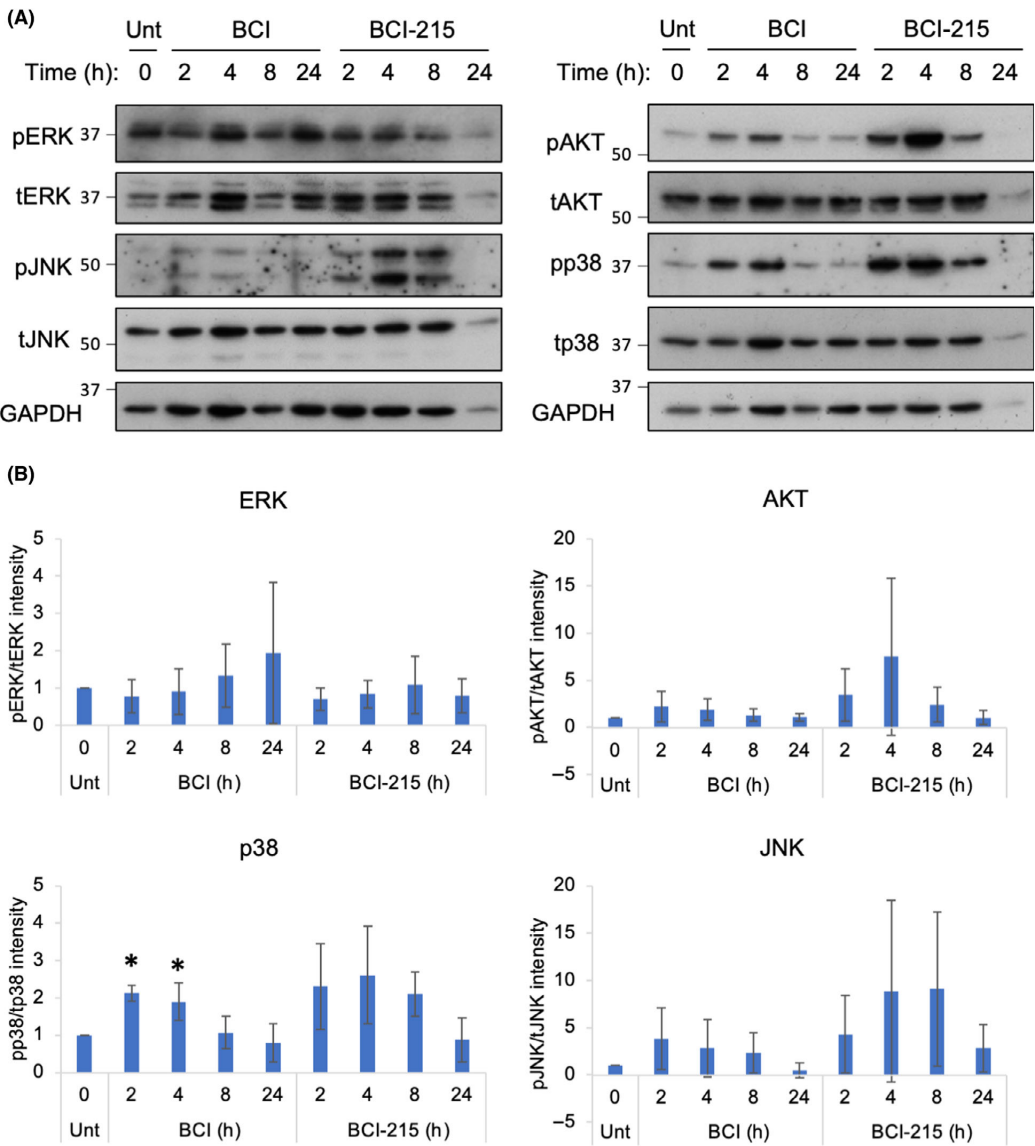
BCI and BCI‐215 induce a short‐lived activation of the stress‐inducible MAPKs. (A) KELLY cells were treated with 2 µm BCI or BCI‐215 for 2, 4, 8 and 24 h and then assessed via an immunoblot for phosphorylated ERK (T185/Y187 (ERK1) or T202/Y204 (ERK2)), JNK (T183/Y185), p38 (T180/Y182) and AKT (S473) (*n* ≥ 3). Each detection is a re‐probe of the same membrane. Molecular sizes of 37 and 50 KDa markers are indicated. (B) For quantification, phosphoproteins were normalised to their respective total proteins, and data were then expressed as a mean ± SD. One‐way ANOVA compared with DMSO; *P ≤ 0.05.

We sought to investigate the biochemical changes induced by BCI and BCI‐215 treatment in the KELLY cells since they had intermediate levels of both *DUSP1* and *DUSP6* gene expression [33]. DUSP6 is regarded as an ERK‐phosphatase that decreases cytosolic levels of pERK, although recently other non‐MAPK‐related substrates have been identified for DUSP6 [34]. DUSP1 is an inducible, nuclear MAP kinase phosphatase (MKP) that dephosphorylates JNK and to a lesser extent p38 and ERK [35]. Surprisingly, treatment with 2 µm BCI did not result in consistent induction of pERK as one would expect from inhibition of DUSP6, although there was an upward trend by 24 h (Fig. 3). Shorter incubation times with BCI were also tested, but again, no reproducible change in pERK was observed (data not shown). In contrast, p38, JNK and AKT were all phosphorylated quickly by both BCI and BCI‐215 within 2 h, indicating a potential role for DUSP1, although this effect was somewhat variable for JNK and AKT. With p38, JNK and AKT, phosphorylation returned to basal levels by 24 h. This acute, short‐lived activation of p38 and JNK signalling could therefore result from cellular stress or direct inhibition of DUSP1. Alternatively, this could suggest the chemical stability of BCI decreases after 4 h.

**Fig. 4:**
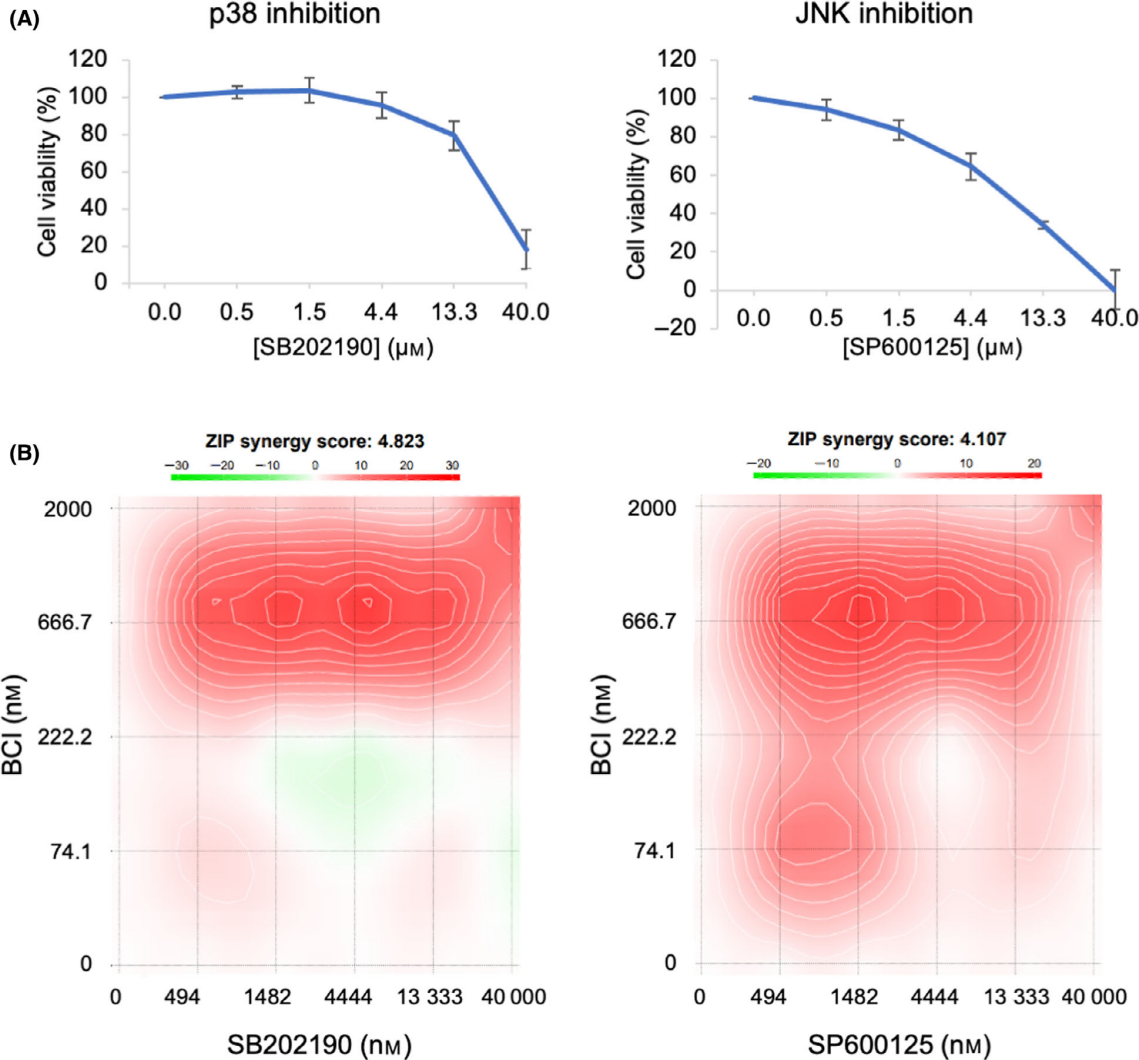
JNK or p38 inhibition does not antagonise BCI‐mediated cell death. (A) KELLY cells were treated with increasing doses of a p38 inhibitor (SB202190) or a JNK inhibitor (SP600125) followed by a cell viability assay. Data were normalised to cells treated with DMSO and then expressed as a mean ± SD (*n* ≥ 3). (B) KELLY cells were treated for 6 days with a range of concentrations of either SB202190 (190) or SP600125 (125) concurrently with BCI, followed by a cell viability assay. Data are presented as synergy plots using the zero interaction potency (ZIP) model. Positive values (red regions) and negative values (green regions) are indicative of synergy and antagonism, respectively. The ZIP synergy score is an average of all the dose combination measurements. Data were generated using synergyfinder (v2.0) [23].

Previous reports suggest pharmacological inhibition of JNK or p38 could attenuate BCI cytotoxicity [17, 20]. To assess this in NB cells, synergy assays were performed by combining BCI with JNK or p38 inhibitors. Inhibition of either JNK or p38 alone had a cytotoxic effect on KELLY cells (Fig. 4A). Both inhibitors demonstrate a mild synergy effect with high concentrations of BCI, but no significant antagonism in any combination (Fig. 4B). These data indicate that JNK and p38 inhibition does not antagonise BCI in these cells.

### Phosphoproteomic analysis of BCI action

**Fig. 5:**
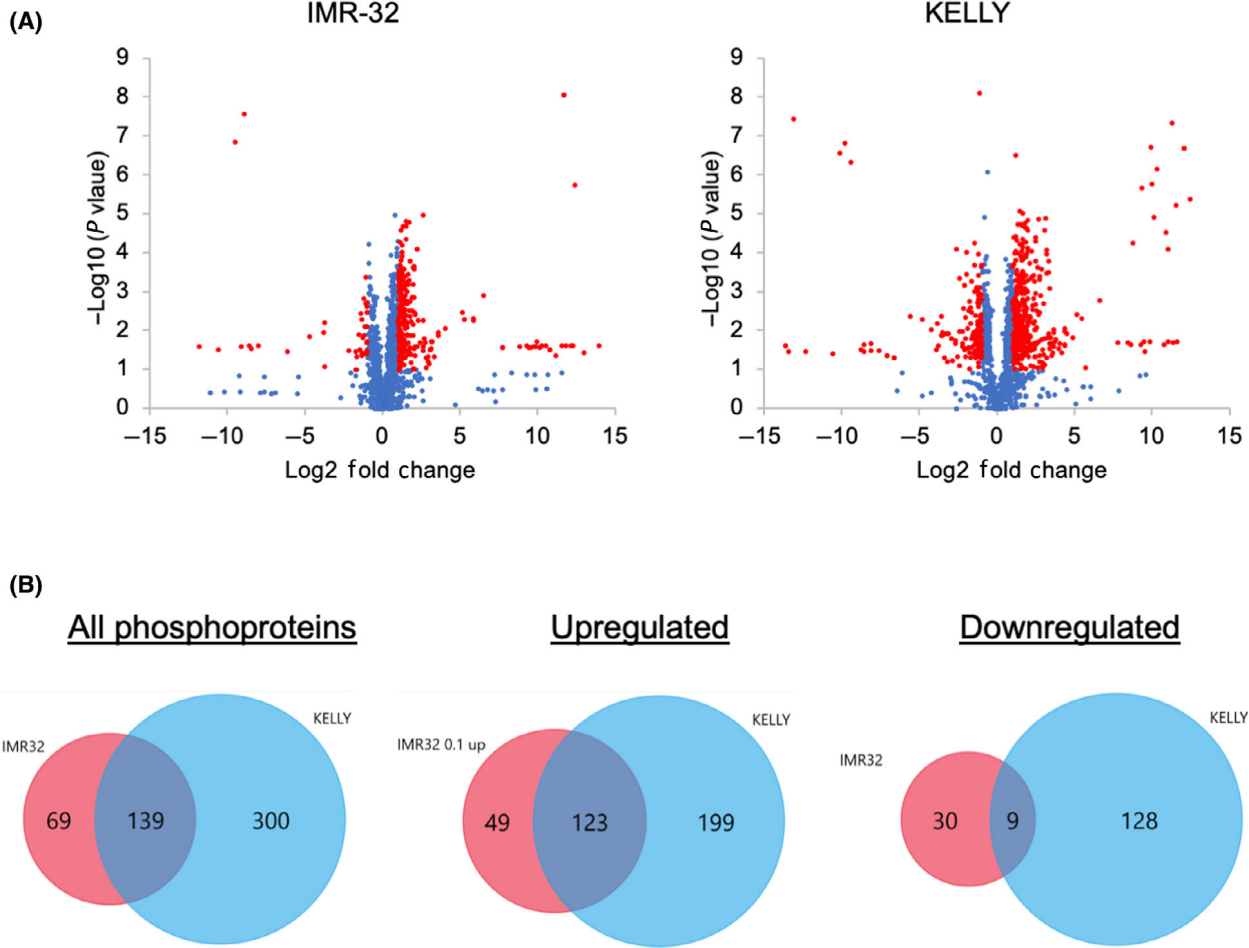
Phosphoproteomic analysis of NB cells after treatment with BCI. (A) Cells were cultured in the presence of 2 µm BCI for 2 h before lysates were collected and subject to pTyr and pSer/Thr phosphoproteomic analysis (*n* = 4). Significant thresholds for differential phosphorylation were set as Log2 fold change > 1 and *P* value < 0.1 (red data points). Volcano plots are shown of KELLY and IMR‐32 cells where each data point represents a phosphosite. (B) Venn diagram of significant phosphoproteins in IMR‐32 and KELLY cells.

**Fig. 6:**
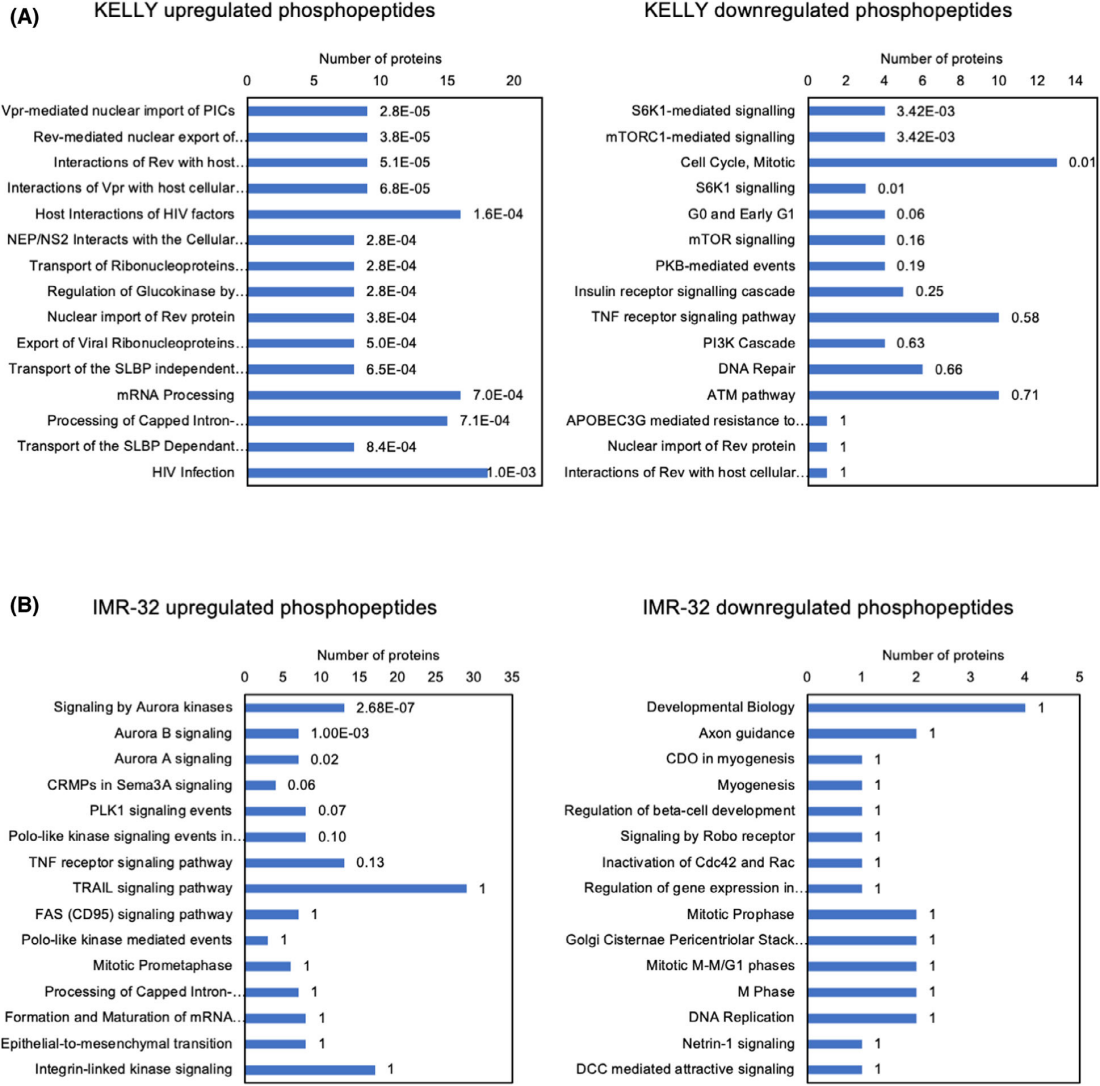
Biological pathway enrichment of phosphoproteome after BCI treatment. Biological pathway enrichment was performed on differentially phosphorylated proteins using the FUNRICH platform. The top 15 differentially upregulated and downregulated pathways in (A) KELLY and (B) IMR‐32 cells are presented with the Bonferroni‐adjusted *P* values adjacent to each bar. Differentially phosphorylated proteins were identified in Fig. 5A (red data points).

**Fig. 7:**
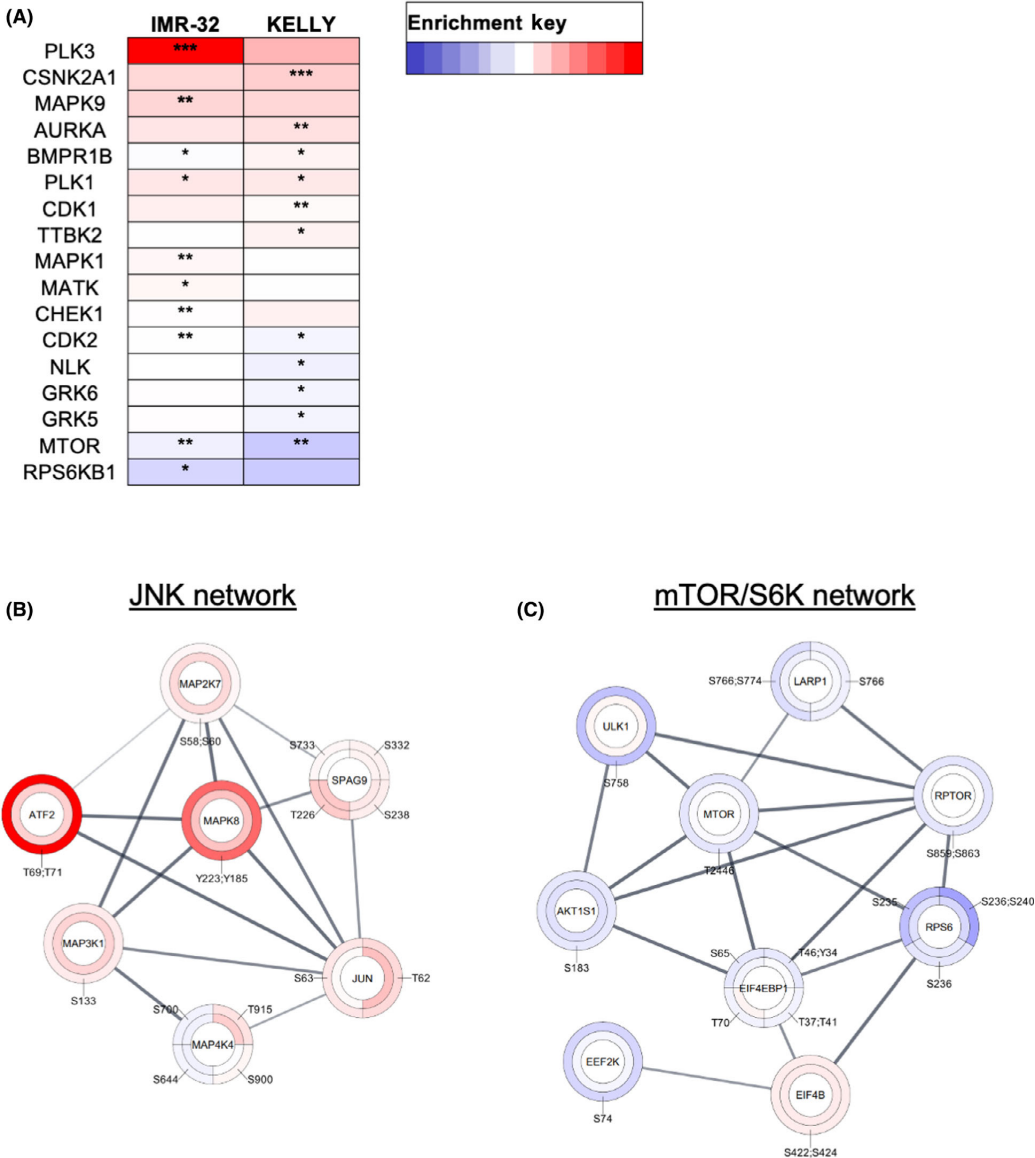
Regulation of JNK and mTOR/R6K signalling pathways by BCI. (A) A KSEA was performed on the phosphoproteomic data. Enriched kinase substrates significant in one or both cell lines are shown. All kinase substrates identified are displayed in Dataset S2. Also shown are networks of proteins related to (B) JNK and (C) mTOR/S6K signalling based on STRING and visualised in Cytoscape. Inner and outer rings represent Log2 fold changes in KELLY and IMR‐32, respectively. Red to blue colour indicates increased and decreased Log2 fold change, respectively, with great colour intensity reflecting greater fold changes.

In various cancer models, treatment with BCI results in an increase in ERK1/2 signalling [18, 19]. However, in KELLY cells BCI induces a transient AKT, JNK and p38 response, during which ERK1/2 activation does not significantly change. We next asked what other MAPK signalling components are activated in NB cells and whether BCI activates non‐MAPK‐related signalling pathways. To achieve this, a phosphoproteomic screen was performed in KELLY and IMR‐32 cells after treatment with 2 µm BCI for 2 h (Dataset S1). Numerous significant changes in phosphopeptides were identified (Fig. 5A). There was a substantial overlap between significant KELLY and IMR‐32 phosphopeptides in response to BCI, and most of this overlap was due to upregulated phosphopeptides (Fig. 5B). A pathway enrichment analysis was performed on the significantly upregulated and downregulated phosphoproteins in IMR‐32 and KELLY cells (Fig. 6). There was a wide range of disparate pathways altered in response to BCI in KELLY cells (Fig. 6A). Of note, mTOR‐ and S6K1‐mediated signalling were significantly enriched by the downregulated peptides in KELLY cells. Only a handful of enriched pathways were identified in the IMR‐32 cells due to the smaller set of 208 phosphopeptides remaining significant after thresholding (Fig. 6B). To determine kinase activity in response to BCI, a kinase substrate enrichment analysis (KSEA) was also performed (Fig. 7A, Dataset S2). In line with our previous findings, the ERK1/2 protein itself was not phosphorylated in response to BCI in either KELLY or IMR‐32. However, the KSEA demonstrated a marginal yet significant upregulation of the ERK1/2 signalling pathway in IMR‐32 cells, likely due to alternative ERK signalling components. As expected, various JNK signalling pathway components were activated including its downstream targets AP‐2 and Jun and its upstream kinases MAP3K1, MAP2K7 and MAP4K4 (Fig. 7A,B). The majority of these JNK‐related phosphopeptides were identified in both KELLY and IMR‐32 cells. The aurora kinase pathways, which have been implicated in NB, also appear differentially regulated in IMR‐32 cells and KELLY cells (*P* = 0.003). However, in both cases, BCI treatment leads to aurora kinase activation, which would be expected to benefit these tumour cells [36]. Examining the kinase substrates suppressed by BCI, it may be of interest that both mTOR signalling and its target S6K are significantly reduced in KELLY and IMR‐32 cells (Fig. 7A). A range of downregulated phosphoproteins related to mTOR and S6K signalling were identified in the KSEA analysis (Fig. 7C). This could relate to the negative survival consequences induced by BCI.

### BCI‐mediated cell death is unaffected by concurrent loss of DUSP1 and DUSP6

**Fig. 8:**
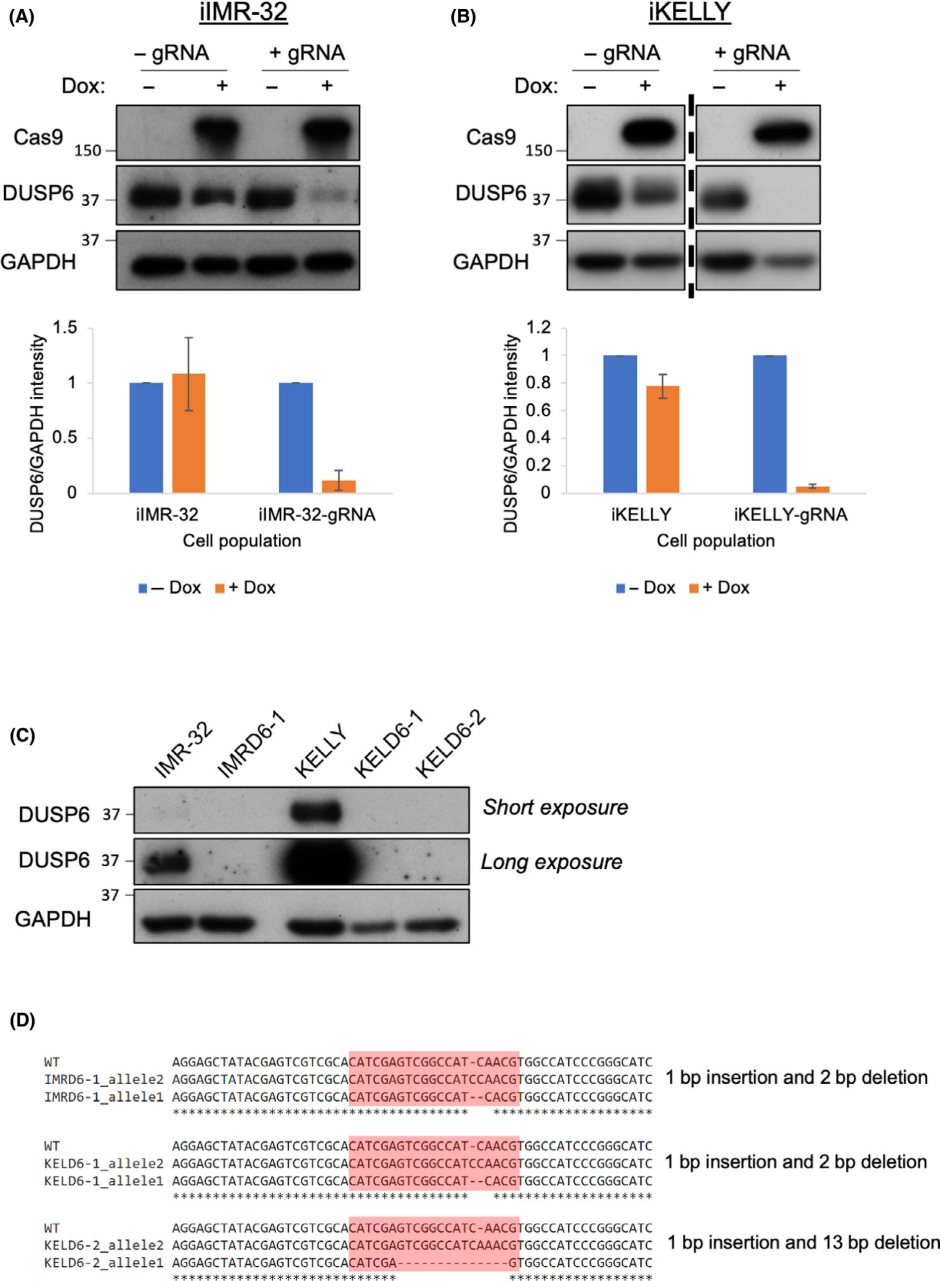
Generation of DUSP6‐null iKELLY and iIMR‐32 cells. (A) IMR‐32 and (B) KELLY cells were transduced with an inducible Cas9 expression vector under the control of a Dox‐inducible promoter, generating iIMR‐32 and iKELLY derivatives. Subsequent transfection of these cells with a DUSP6 gRNA and treatment with Dox resulted in a significant depletion of DUSP6 protein in the overall populations. Cells were treated with 0.5 µg·mL^−1^ Dox for 4–9 days (*n* = 3). Dividing line in B separates the identical exposure of two sections of the same membrane. For quantification, DUSP6 protein was normalised to GAPDH, and data were then expressed as a mean ± SD. (C) Removal of DUSP6 protein expression via CRISPR‐Cas9 was confirmed using immunoblots in two separate, single‐cell cloned sublines from iKELLY (KELD6‐1 and KELD6‐2) and one of iIMR‐32 cells (IMRD6‐1). (D) Sequence alignments of a region of the wild‐type DUSP6 sequence alongside both alleles in KELD6‐1 and KELD6‐2 and in IMRD6‐1 subclones. Highlighted in red is the DUSP6 gRNA sequence and the indels present in each subclone are displayed next to each alignment. Each vertically stacked set of immunoblots shows detections after reprobing the same membrane.

Considering that inhibition of both DUSP1 and DUSP6 was not greatly affecting ERK signalling, we became concerned that BCI was inducing cell death via unrelated, off‐target toxicity, potentially based on nonspecific activation of the stress‐induced JNK and p38 MAPK. To investigate whether cytotoxicity induced by BCI was mediated via DUSP6, indels were introduced into the *DUSP6* gene in KELLY and IMR‐32 cells to completely deplete functional DUSP6 protein. To this end, a tetracycline‐inducible CRISPR‐Cas9 system was developed in these cells to generate iIMR‐32 and iKELLY derivatives where Cas9 was inducible by Dox (See Materials and methods; Fig. 8A,B). Treating iIMR‐32 and iKELLY cells with a DUSP6 gRNA plasmid and Dox resulted in significant depletion of DUSP6 protein. Single‐cell serial dilution generated subclones IMRD6‐1, KELD6‐1 and KELD6‐2 with complete loss of DUSP6 as evidenced by long exposures of immunoblots (Fig. 8C). The individual subclones used for further analysis contained compound heterozygous deletions in the DUSP6 gene (Fig. 8D).

**Fig. 9:**
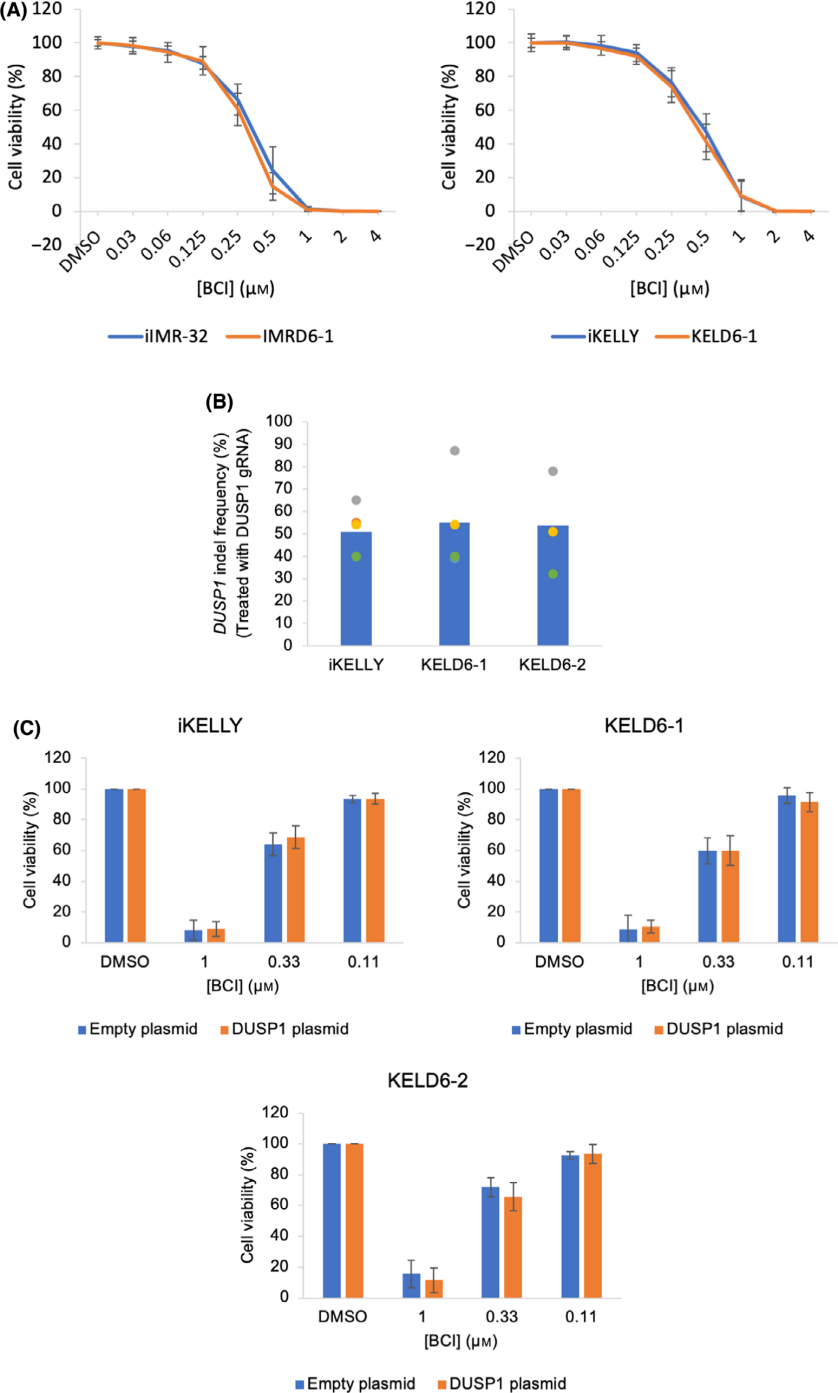
Loss of DUSP1 and DUSP6 does not attenuate BCI‐mediated cell death. (A) IKELLY and iIMR‐32 cells and DUSP6‐null subclones IMRD6‐1 and KELD6‐1 were treated with increasing doses of BCI, and cell viability assays were performed. An independent‐samples *t*‐test was performed at ~EC50 values (0.5 and 0.25 µm for iKELLY and iIMR‐32 cells, respectively) and was not significant. (B) ICE analysis of mixed populations, showing indel frequencies in the *DUSP1* gene in iKELLY, KELD6‐1 and KELD6‐2 after transfection with the DUSP1 gRNA (*n* = 3). (C) Derivatives of iKELLY wild‐type cells and DUSP6‐null subclones KELD6‐1 and KELD6‐2 containing (i) mixed population indels in the *DUSP1* gene or (ii) control populations treated with empty, negative control plasmid, were subject to BCI treatment and a cell viability assay. Data were normalised to cells treated with DMSO and then expressed as a mean ± SD (*n* ≥ 3). A one‐way ANOVA was performed and comparisons between equivalent BCI treatments showed no significant changes.

The cytotoxicity of BCI was investigated in DUSP6‐null KELLY and IMR‐32 cells. Surprisingly, complete loss of DUSP6 protein did not improve the survival of iKELLY or iIMR‐32 cells in response to BCI, suggesting BCI‐induced cell death does not rely on DUSP6 inhibition (Fig. 9A). To determine whether cell death was via DUSP1 inhibition, iKELLY cells and DUSP6 knockout subclone cells KELD6‐1 and KELD6‐2 were then further depleted of DUSP1 protein. To achieve this, iKELLY cells and DUSP6‐null subclones were transfected with a DUSP1‐specific gRNA and subject to transient Cas9 expression. Since the DUSP1 protein expression could not be detected in an immunoblot (data not shown), the indel frequency in the *DUSP1* gene was used instead as an indirect measure of functional DUSP1 protein. The genomic DNAs from targeted cell populations were sequenced and demonstrated > 50% out‐of‐frame indels (Fig. 9B). In Fig. 9C, the DUSP1‐treated samples were mixed populations with respect to DUSP1 indels, but monoclonal with respect to the DUSP6 indels in KELD6‐1 and KELD6‐2. Similar to loss of DUSP6, partial depletion of the DUSP1 protein in cells with or without DUSP6 does not affect the efficacy of BCI in iKELLY cells (Fig. 9C). Although indel frequencies were variable between experiments, in individual cases where > 80% of the population contained out‐of‐frame indels (Fig. 9B), there remained no attenuation of BCI cytotoxicity. This is a challenging result and suggests that BCI‐mediated cell death in these particular cell lines is a result of off‐target toxicities and not due to the chemical inhibition of DUSP1 or DUSP6.

## Discussion

High‐risk NB remains a paediatric disease with a dismal prognosis for many patients. Due to the frequent lack of targetable aberrations, the treatment regimen is very intense and nonspecific, hence it is imperative that new therapeutic options are identified. Given the documented evidence of DUSP enzymes having pro‐oncogenic activities in various cancers [37, 38], we have investigated the therapeutic potential of BCI, a dual DUSP1/6 inhibitor, across a range of NB cell lines. Like what has been observed in other cancer models, BCI demonstrated cytotoxicity in all NB cells investigated. Phosphoproteomic analysis identified a wide range of upregulated pathways including the stress‐activated JNK kinase. It also suppressed potential survival pathways through mTOR and S6K. Disappointingly, depletion of either target protein DUSP1 or DUSP6, or both together, did not influence the potency of BCI in the context of KELLY and IMR‐32 NB cells. Taken together, this indicates that BCI unfortunately generates significant off‐target toxicity effects in NB cells and that its actual targets remain to be determined.

Initial evidence in SH‐SY5Y cells demonstrated that BCI induces cell death in a dose‐dependent manner [21]. This is in accord with a range of other studies where BCI causes cancer cell cytotoxicity via apparent inhibition of DUSP1 or DUSP6 [18, 19, 20]. In the present study, BCI is also very cytotoxic in NB cells with EC50 values in the low micromolar range. ERK is regarded as the major substrate of DUSP6, with key interactions at the ERK‐DUSP6 interface being required for DUSP6 phosphatase activity [39]. It was therefore surprising that BCI did not induce any significant alterations in ERK phosphorylation in the NB cells. This is in contrast to a previous report where BCI treatment increased pERK signalling in SH‐SY5Y cells [21]. Considering experiments here were performed in KELLY cells, this discrepancy may be due to variable intracellular signalling between NB cell lines. Furthermore, in ovarian cancer cells, treatment with BCI elicited a strong upregulation of pERK and ERK pathway response genes [40]. Also, a phosphokinase screen in breast cancer cells demonstrated that BCI‐215, a BCI analogue, induced the ERK, JNK and p38 pathways, but crucially had little effect on non‐MAPK‐related pathways [17]. Thus, although we did not observe ERK activation in NB cells, we did find changes in phosphorylation of JNK and p38 and also changes in a wide range of non‐MAPK‐related signalling pathways, including mTOR and its target S6K. In contrast to these studies, and like our own observations, a transcriptional study investigating osteoclast differentiation after BCI treatment did not identify the MAPK pathways. Instead, there was a significant downregulation of the STAT3 and NF‐κB pathways [41]. This wider effect on the intracellular phosphatome by BCI could be suggestive of much broader inhibition of DUSP phosphatases or, instead, other unrelated enzymes. Molecular docking simulations demonstrated BCI binds to DUSP1/6 in an allosteric manner, preventing a conformational change that normally activates its phosphatase activity [16]. It is unlikely therefore that other phosphatases offer the same binding pocket for BCI. Indeed, BCI did not inhibit recombinant CDC25B, PTP1B or DUSP3 in vitro [15]. Nonetheless, as numerous DUSPs are important regulators of neuronal cell growth, differentiation and apoptosis, it is still possible BCI is inhibiting other DUSPs in NB cells. It would be beneficial if a larger analysis was performed on BCI‐binding affinity to other phosphatases [8].

Unfortunately, at least in NB cells, DUSP1 and DUSP6 both appear dispensable for BCI‐mediated cell death. If this is not via DUSP1 or DUSP6 inhibition, then the cytotoxic mechanism of BCI needs to be further elucidated. We demonstrate in NB cells that BCI triggers both p38 and JNK signalling, possibly transiently. We also demonstrate that over the first 24 h of treatment there is an increase in apoptotic signalling. Other studies have demonstrated the role of JNK signalling in BCI‐mediated cell death. In malignant peripheral nerve sheath tumour cells, cell death via BCI was antagonised by JNK inhibition [20]. Furthermore, in breast cancer cells, inhibition of the p38 but not JNK or ERK partially rescued cell death from the BCI analogue BCI‐215 [17]. Although neither case completely abrogated BCI cytotoxicity, these pathways clearly play an important role of BCI function in those cells. In NB cells, however, the situation is more complex since the inhibition of JNK or p38 themselves decreases cell viability. Nonetheless, even at lower concentrations of JNK and p38 inhibition, the cytotoxicity of BCI is not attenuated. This leaves the possibility that DUSP1 inhibition by BCI is still contributing to the JNK activation in KELLY and IMR‐32 cells, as it does in other cancer cell types [19, 20], but this is not the cytotoxic effector of BCI.

The inactivation of the mTOR‐S6K axis by BCI might suggest that BCI inhibits a target in this pathway. This is supported by a study in pre‐B acute lymphoblastic leukaemia cells where BCI treatment inhibited RPS6 phosphorylation within 2 h [19]. In our study, as AKT was acutely upregulated by treatment with BCI, this response is clearly more complex than suppression of the canonical PI3K/Akt/mTOR pathway. Nonetheless, inhibition of mTOR reduces NB cell proliferation [42], therefore this potential function of BCI warrants further study in these cells. Treatment with BCI and concurrent activation of the stress‐inducible MAPKs consistently leads to the accumulation of reactive oxygen species (ROS) [17, 18, 19]. Oxidation of the catalytic cysteine is well known to inhibit the enzymatic activity of tyrosine phosphatases, including that of DUSP6 [43, 44]. If ROS were to play a role in BCI action, this may result in broad, nonspecific inhibition of PTP enzymes. In HeLa cells, TNFα‐induced ROS resulted in significant inhibition of various DUSPs, activation of JNK and necrotic cell death [45]. As TNF signalling was apparently upregulated in IMR‐32 cells, BCI could have activated this pathway to mediate ROS production. In pre‐B acute lymphoblastic leukaemia cells, BCI‐mediated cell cytotoxicity was partially rescued by ROS scavengers, demonstrating a possible mechanism of cell death [19]. ROS effects of BCI could therefore be examined in NB cells in the future.

Historically, target specificity has hampered the development of phosphatase inhibitors [46]. Previously identified DUSP1/6 inhibitors such as NSC‐95397 tend to demonstrate a binding affinity towards additional phosphatases [47, 48]. These challenges appeared to be overcome by BCI as it binds allosterically to DUSP1 and DUSP6. Unfortunately, this is not the straightforward case in NB cells where BCI cytotoxicity is independent of its stated DUSP targets. Similar results have been observed by active‐site SHP2 inhibitors, which upregulated PDGFRβ signalling in SHP2 knockout cells [49]. *In vitro* enzymatic assays demonstrated some inhibitors targeted PDGFRβ directly, whereas others function indirectly on PDGFRβ. These results demonstrate how caution must be used when using pharmacological inhibitors to probe the molecular role of a phosphatase target. However, off‐target toxicity appears a broader issue in cancer drug development [50, 51]. Lin et al. [50] performed a CRISPR‐wide screen to validate the on‐target effect of a panel of drugs in clinical or preclinical trials. Strikingly, a wide range of cancer drugs displayed cytotoxicity in cells depleted of their molecular target.

In conclusion, the results presented demonstrate that BCI, a putative DUSP1/6 inhibitor, is cytotoxic in a range of NB cell lines and induces a short‐lived activation of the AKT and stress‐inducible MAP kinases. Loss of DUSP1 or DUSP6, however, did not reduce the efficacy of BCI. Thus, the cytotoxic effect of compounds like BCI must be validated rigorously in either gene knockout models of both DUSP1 and DUSP6 or with other protein inactivation approaches. Although BCI has not proven of good specificity in NB cell lines, the chemical does demonstrate cytotoxicity in a range of other cancer models, and in those cases, BCI may still be showing stronger on‐target and weaker off‐target actions. While the targeting of DUSP1 and DUSP6 is not sufficient to explain the cytotoxicity of BCI in NB cells, its key cytotoxic targets remain to be determined. Our current data indicate that its effector pathway may include proteins in the mTOR/S6K pathway, and this would warrant further study.

## Conflict of interest

The authors declare no conflict of interest.

## Author contributions

ET, VP and AWS conceived and designed the project. ET, VP and VR acquired the data, with VR and PRC providing the proteomics platform. All authors contributed to the analysis and interpretation of the data. ET and AWS wrote the paper with input from the other authors.

## Supporting information


**Dataset S1.** Phosphopeptide changes in KELLY and IMR‐32 cells after treatment with BCI. Raw phosphoproteomic values for peptides were converted to Log2 fold change compared with DMSO treatment. Alongside Log2 fold change is a p value of the differences (assessed by t‐test of log‐transformed data assuming unequal variances).

**Dataset S2.** KSEA results generated using phosphoproteomic datasets. KSEA was performed on IMR‐32 and KELLY dataset as described in [23]. The z‐score represents the distribution of changes in phosphorylation of substrates for given kinases relative to the distribution of all fold changes. Each row displays a kinase substrate alongside the substrates related to that kinase identified in the phosphoproteomic dataset. The z‐score is colour‐coded as blue (decreased) or red (increased) with asterisks denoting the significance of differences.

## Data Availability

The additional data that support the findings of this study are available either in associated supplementary files or from the corresponding author a.stoker@ucl.ac.uk upon reasonable request. The latter includes the raw phosphoproteomics data output.
